# Sustainable Manufacturing of Lightweight Hybrid Nanocomposites for Electric Vehicle Battery Enclosures

**DOI:** 10.3390/polym17081056

**Published:** 2025-04-14

**Authors:** Umar Farooq, Valentina Bertana, Giulia Mossotti, Sergio Ferrero, Luciano Scaltrito

**Affiliations:** 1 Chilab-ITEM Laboratory, Department of Applied Science and Technology (DISAT), Politecnico di Torino, Corso Duca degli Abruzzi 24, 10129 Turin, Italy; valentina.bertana@polito.it (V.B.); giulia.mossotti@polito.it (G.M.); sergio.ferrero@polito.it (S.F.); luciano.scaltrito@polito.it (L.S.); 2 Department of Science and Technology Innovation (DISIT), Università del Piemonte Orientale, Viale Teresa Michel 11, 15121 Alessandria, Italy

**Keywords:** hybrid CFRP, epoxy nanocomposites, multiwalled carbon nanotubes, ionic liquid, mechanical properties, electrical conductivity, thermal management, sustainable automobile applications, battery pack enclosures, vacuum bag process

## Abstract

Nanocomposite laminates containing carbon fibers, epoxy, and multiwalled carbon nanotubes were fabricated using a vacuum bag process. Ecofriendly ionic liquid (5 wt%)-treated multiwalled carbon nanotubes (pristine and nickel-coated) were added to the epoxy independently, in amounts ranging from 1 wt% to 3 wt%, in order to tailor the mechanical, electrical, and thermal performance of manufactured carbon fiber epoxy composite laminates. These nanocomposite laminates were later characterized through flexural testing, dynamic mechanical analysis, impedance spectroscopy, thermal conductivity tests, and FTIR spectroscopy to evaluate their suitability for battery pack applications. The findings showed that both types of multiwalled carbon nanotubes exhibited multifaceted effects on the properties of bulk hybrid carbon fiber epoxy nanocomposite laminates. For instance, the flexural strength of the composites containing 3.0 wt% of ionic liquid-treated pristine multiwalled carbon nanotubes reached 802.8 MPa, the flexural modulus was 88.21 GPa, and the storage modulus was 18.2 GPa, while the loss modulus peaked at 1.76 GPa. The thermal conductivity of the composites ranged from 0.38869 W/(m · K) to 0.69772 W/(m · K), and the electrical resistance decreased significantly with the addition of MWCNTs, reaching a minimum of 29.89 Ω for CFRPIP-1.5 wt%. The structural performance of hybrid nanocomposites containing ionic liquid-treated pristine multiwalled carbon nanotubes was higher than that of the hybrid nanocomposite of ionic liquid-treated Ni-coated multiwalled carbon nanotubes, although the latter was found to possess better functional performance.

## 1. Introduction

Carbon fiber-reinforced hybrid nanocomposites (CFRPs) are extensively employed in the aerospace, automotive, and sporting goods sectors due to their superior structural and functional performance [1,2,3]. These composites show significant improvements in strength, heat resistance, and electrical conductivity, making them ideal choices for manufacturers of battery cases and for other relevant applications in automobile sectors [4,5,6]. For example, Ren et al. [4] reported a tensile strength of 1200 MPa for CFRPs under corrosive conditions, highlighting their durability, while Moyer et al. [5] achieved a thermal conductivity of 0.8 W/(m · K) in carbon fiber-reinforced structural batteries, emphasizing their thermal management potential. Further, the versatility of CFRP composites allows for tailored properties through variations in fiber orientation, fiber treatment, and matrix modification using various weight fractions of nanomaterials to optimize the designs for specific applications [7,8,9]. The exploration of various combinations and methods in manufacturing CFRPs is essential for uncovering new opportunities, particularly due to the rising demand for lightweight and durable materials for sustainable mobility. Therefore, industries are focusing on CFRP hybrid composite applications in emerging technologies, aiming to improve performance and support sustainability by lowering energy consumption and emissions [10,11,12,13,14].

The manufacture of hybrid CFRPs involves the incorporation of different types of nanomaterials into the matrix to significantly enhance the properties and functionalities of the final composites [15,16]. These nanomaterials include nanoparticles, nanotubes, and nanofibers, which are utilized to improve the mechanical strength, thermal stability, electrical conductivity, and barrier properties of the composites [17,18]. The incorporation of carbon nanotubes (CNTs), such as multiwalled carbon nanotubes (MWCNTs), in CFRPs improves the structural and functional performance, specifically when these MWCNTs are treated/coated with ecofriendly materials to enhance their dispersion in the polymer matrix, making them suitable for high-performance applications, especially sustainable automobile applications [19,20,21].

In particular, Wu et al. [19] demonstrated that the addition of 1 wt% functionalized MWCNTs to CFRPs increased the tensile strength by 30% to approximately 1500 MPa and improved the thermal conductivity to 1.2 W/(m · K), signifying the potential use of MWCNTs as reinforcing nanomaterials. Dhanaraju et al. [22] reported that adding 1.0 wt% silane-functionalized MWCNTs improved flexural strength by 73.8% and modulus by 58.2%. Similarly, Brown et al. [23] found that the presence of MWCNTs at interlayers increased the loss modulus by 182% and storage modulus by 2.2%. An increase in thermal conductivity was also reported by Kumar et al. [24], who achieved 5.8 W/(m · K) at 0.5 wt% MWCNTs, and Chen [25], noting 10.2 W/(m · K) at 1.0 wt%. In addition, regarding electrical performance, Spinelli et al. [26] observed a significant decrease in resistance at 0.5 wt% of carbon nanotubes, and Zhang et al. [27] observed a substantial decrease in resistance at 0.2 wt%, highlighting the significance of the incorporation of MWCNTs in advanced hybrid nanocomposites.

Different types of coating materials are employed on the surface of CNTs to improve their dispersion in polymer matrices and to facilitate interfacial adhesion with the matrix, as well as with reinforcement fibers in the final manufactured composites [28,29,30,31]. These coatings include polymeric [32], metallic layers [33], and silane [30], each tailored to enhance their compatibility with the specific polymer matrix in order to optimize the performance of the manufactured composites for their final applications. Additionally, ongoing research is also focusing on the evaluation of the long-term stability and environmental impact of these modified MWCNTs, considering their costs and sustainability [34].

In addition to the application of coating materials on the surface of MWCNTs, researchers have functionalized MWCNTs using various approaches [35]. The functionalization of MWCNTs greatly improves nanotube surfaces by adding different chemical species, which increase their reactivity and compatibility with polymeric materials, resulting in significant enhancements in the electrical, mechanical, and thermal properties of composite materials manufactured for various applications [36]. To functionalize MWCNTs, researchers have used acids [37], plasma [38,39], alkalis [40], ecofriendly solvents [41], surfactants [42], organic compounds [43], and so on. These studies suggest that while functionalization methods such as acid treatments, thermal decomposition, and plasma treatments effectively enhance the surface properties of MWCNTs, they often compromise the structural integrity of MWCNTs to achieve enhanced functionalization [30,44,45].

The scalability of these methodologies is also important within industrial contexts, as this directly influences feasibility and efficiency at the bulk level. Consequently, ecofriendly techniques for the treatment of MWCNTs are becoming more popular because of their low environmental impacts, recyclability, and simple processing while delivering optimum results [46,47,48]. Ecofriendly solvents’ characteristics have opened up their potential in new domains, such as renewable energy, electronics, and biomedicine, where customized materials may enhance performance and promote sustainability [47,48]. Ecofriendly solvents include various natural extracts, ionic liquids (ILs), and biodegradable polymers, which not only facilitate the functionalization process but also minimize toxic byproducts [49,50,51].

Due to their tunable chemical species (cations and anions) and simple processing, ILs have emerged as effective and ecofriendly materials for treating various nanomaterials to enhance their interaction with the polymer matrix and obtain desired structural and functional properties [52,53,54]. Due to the low evaporation and adjustable solvation properties of ionic liquids, researchers have found that IL treatment not only facilitates a uniform dispersion of nanomaterials in polymer matrices but also improves the overall electrical, mechanical, and thermal properties of the system [54]. Therefore, this ecofriendly treatment method contributes to the advancement of nanotechnology, in line with the growing concern regarding sustainability [55].

Furthermore, the integration of IL-modified carbon nanotubes into polymer matrices can significantly improve mechanical strength and thermal stability, paving the way for next-generation materials that are lighter, more durable, and more efficient. Different types of ILs can be tailored to achieve specific interactions with carbon nanotubes, allowing for the fine-tuning of their properties and performance in diverse applications. The currently available ILs exhibit a wide range of characteristics, including varying degrees of hydrophobicity and viscosity, which can be strategically selected based on the desired outcome in the final composite [56].

Imidazolium-based ILs have shown an optimum ability to enhance the dispersion and stability of carbon nanotubes within the polymer matrix, ultimately leading to composites with superior electrical conductivity and mechanical performance [57]. Moreover, the incorporation of ILs can also facilitate the processing of the composites at lower temperatures, reducing energy consumption and preserving the integrity of the carbon nanotubes.

In the current study, two types of MWCNTs—namely, pristine MWCNTs and nickel-coated MWCNTs—were treated with 5 wt% of imidazolium-based IL (BMI-TFSI) to examine their effects on the structural and functional properties of CFRPs at different weight fractions (1.0 wt%, 1.5 wt%, 2.0 wt%, 2.5 wt%, and 3 wt%) in two batches, separately. A reference CFRP without MWCNTs was also prepared for the comparison and comprehensive analysis of the enhancements in the structural and functional performance of the manufactured modified CFRPs. The results indicate that the incorporation of IL-treated MWCNTs significantly improved the properties of CFRPs, highlighting the importance of an ecofriendly process for the surface modification of MWCNTs to enhance the interfacial bonding within the CFRP matrix. Despite the extensive literature on MWCNT-reinforced CFRPs, a gap remains in understanding the comparative effects of ecofriendly ionic liquid treatments on various nanomaterials. The primary objective of this study was to investigate the effects of ionic liquid-treated pristine and nickel-coated MWCNTs on the mechanical, electrical, and thermal properties of CFRPs, aiming to identify optimal formulations for battery pack enclosures. Moreover, this work signifies the critical need for lightweight, durable, and thermally efficient materials that enhance safety and performance in sustainable mobility applications, where existing materials often compromise structural integrity and functional performance.

## 2. Materials and Methods

### 2.1. Materials

The materials used to fabricate CFRPs with and without the incorporation of IL-treated carbon nanotubes comprised the following: (i) polyacrylonitrile (PAN)-based 3K-plain weave (0°, 90°) carbon fiber fabric (Toray, Taipei, Taiwan); (ii) epoxy resin (Araldite® 5052, Huntsman, The Woodlands, TX, USA) with epoxy hardener (Aradur® 5052, Huntsman, The Woodlands, TX, USA); (iii) pristine MWCNTs (P-MWCNTs) with an outer diameter of 20–40 nm and length of 5–15 μm, procured from Chengdu Intl, Chengdu, China; (iv) nickel-coated MWCNTs (Ni-MWCNTs) with an outer diameter of 5–15 nm, length > 50 μm, MWCNT content >38 wt%, and Ni content >60 wt%, procured from US Research Nanomaterials Inc., Houston, TX, USA; (v) an ionic liquid, 1-butyl-3-methylimidazolium bis(trifluoromethyl sulfonyl)imide, which is denoted as [BMIM][Tf_2_*N*] or BMI-TFSI, with 98% purity (CAS: 174899-83-3), procured from Sigma-Aldrich, Darmstadt, Germany; (vi) ethanol with 99.5% purity, procured from Sigma-Aldrich, São Paulo, Brazil; (vii) a vacuum bagging kit procured from Easy Composites, Stoke-on-Trent, UK, which was used to obtain compact laminates under vacuum, with and without the incorporation of MWCNTs.

### 2.2. Methods

**Figure 1:**
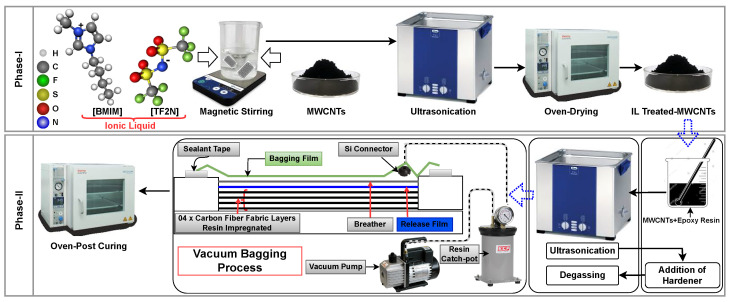
Schematic diagram of the manufacture of hybrid nanocomposites (CFRPs).

The fabrication of the CFRPs was divided into two phases as shown in Figure 1. In the first phase, the surface treatment of both types of MWCNTs (P-MWCNTs and Ni-MWCNTs) with IL was performed, and in the second phase, these IL-modified MWCNTs were incorporated into the epoxy matrix, followed by the impregnation of the carbon fiber fabric with the modified epoxy matrix.

#### 2.2.1. Phase I: Surface Treatment of MWCNTs

In the first phase, both types of MWCNTs, i.e., P-MWCNTs (3 g) and Ni-MWCNTs (3 g), were treated with an IL called BMI-TFSI (5 wt% with respect to the weight fraction of MWCNTs), separately. Initially, BMI-TFSI (5 wt%, 150 mg) was added to ethanol (50 mL) in a beaker, and the solution was stirred for 10 min at room temperature using a magnetic stirrer. P-MWCNTs (3 g) were then added to the beaker containing the ethanol (50 mL) and BMI-TFSI (150 mg) for stirring at room temperature for 15 min. Then, sonication was performed for 30 min at 60 °C and 60 Hz to reduce agglomerates. After sonication, the mixture was dried using a vacuum oven at 60 °C for 1 h to remove the ethanol and to obtain the P-MWCNTs treated with BMI-TFSI in powder form. The same process was repeated for Ni-MWCNTs to obtain BMI-TFSI-treated Ni-MWCNTs in powder form.

#### 2.2.2. Phase II: Fabrication of CFRP Laminates

In the second phase, the dried MWCNTs (P-MWCNTs and Ni-MWCNTs) treated with 5 wt% of IL were added separately to epoxy matrix using 1 wt%, 1.5 wt%, 2.0 wt%, 2.5 wt%, and 3.0 wt% of IL-treated P-MWCNTs and Ni-MWCNTs to fabricate two different batches of composite laminates, referred to as CFRPIP and CFRPINi, respectively. Initially, MWCNTs were mixed into epoxy resin by hand stirring for 5 min, followed by sonication for 45 min at room temperature, ensuring a uniform dispersion in the epoxy matrix. In addition, the sonication time for high-weight fractions (3.0 wt%) of P-MWCNTs and Ni-MWCNTs was increased from 45 min to 60 min to facilitate dispersion. After sonication, hardener with a predefined weight ratio was added to the mixture, which was mechanically mixed by hand stirring again. To remove the bubbles, the mixture was degassed for 10 min in an ultrasonicator.

The resulting modified epoxy matrix was then applied to 4× layers of carbon fiber fabric on a mold, followed by a vacuum bagging process, and cured at room temperature for 24 h. After this curing, the laminates were removed from the mold and post-cured at 100 °C for 4 h using an oven. A reference CFRP laminate without MWCNTs was also manufactured using the vacuum bagging technique to compare its structural and functional performance with that of the two abovementioned batches of composite laminates containing IL-treated P-MWCNTs and Ni-MWCNTs.

The composite laminates were then subjected to various tests, including flexural tests, dynamic mechanical analysis (DMA), impedance spectroscopy, thermal conductivity tests, scanning electron microscopy (SEM), and Fourier-transform infrared spectroscopy (FTIR) assessments, to evaluate their performance.

### 2.3. Characterization

Flexural testing was performed on a universal testing machine (UTM), Galdabini (Galdabini, S.p.a, Cardano al Campo (VA), Italy) using a 30 kN load cell. Five specimens (60 × 13 × 1 mm) of each composite were tested, selecting a cross-head speed of 2 mm/min according to ASTM D7264 [58]. Later, ANOVA was carried out on the resultant flexural strength and flexural modulus test results using Origin Pro 2021. Tukey tests with a 5% significance level were used to compare the groups to evaluate any statistically significant differences among them.

ASTM D7028 [59] was followed to perform DMA tests to evaluate the viscoelastic properties of the manufactured composites. The specimens were cut to size (60 × 12 × 1 mm) and analyzed using DMA7100 equipment (Hitachi Ltd., Hitachi, Japan) in dual cantilever mode and with a strain amplitude of 1 Hz. The temperature range (25–250 °C) was selected to run the scan at a heating rate of 10 °C/min and at a constant frequency of 1.0 Hz to measure the storage modulus ($E^{\prime }$), loss modulus ($E^{\prime \prime }$), and tan δ values. The Tg was observed from the tan δ data.

Impedance spectroscopy (Zurich Instruments, Zurich, Switzerland) was conducted to evaluate the electrical properties of the epoxy samples, measuring the complex impedance over a frequency range of 10 Hz to 1 MHz. The samples were prepared in a parallel plate configuration, and the data were analyzed to determine the dielectric constant and conductivity, providing insights into the material’s performance under varying conditions.

The thermal conductivity (Tc) was measured using Thermtest model apparatus GHFM-01 (Thermtest Inc., Fredericton, NB, Canada) using ASTM E1530-19 [60]. First, the equipment was calibrated using standard 03 × SS 304 samples that had 50 mm diameters and thicknesses of 1, ½, and ¼ inches. The calibration temperatures were selected considering the operational conditions of the target application (i.e., battery-pack housing). The selected temperatures were 40 °C, 60 °C, and 90 °C, keeping the temperature variation between the upper and lower plates within 30 °C (+15 °C and −15 °C) for each temperature value used.

SEM images were captured using a Phenom XL G2 (Thermo Fisher Scientific, Waltham, MA, USA) with the following parameters: 10 kV HV, a BSD Full detector, a 0.10 Pa vacuum, a working distance (WD) of 10–11 mm, a 30 μm aperture, and an InLens signal.

Fourier-transform infrared spectroscopy (FTIR) was performed with a PerkinElmer Spectrum Two FT-IR spectrometer (PerkinElmer Inc., Shelton, CT, USA). The transmission spectra were recorded with KBr pellets in the 400–4000 cm^−1^ range with 32 scans at a constant spectral resolution of 4 cm^−1^, using a LiTaO_3_ detector.

## 3. Results and Discussion

**Figure 2:**
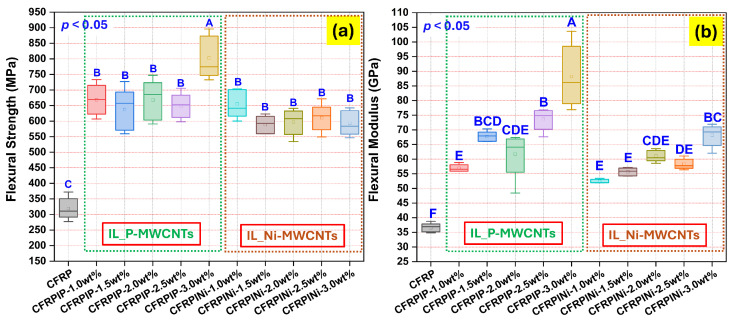
The flexural properties of carbon fiber-reinforced hybrid nanocomposites (CFRPs): (**a**) flexural strength of tested samples; (**b**) corresponding flexural modulus values.

Figure 2 shows two box-and-whisker plots for the flexural strength and flexural modulus representing the distribution of the data across various types of hybrid CFRPs, with and without IL-treated MWCNTs. The data highlight the effects of varying weight fractions of MWCNTs (P-MWCNTs and Ni-MWCNTs) on the mechanical performance of the CFRPs. Moreover, statistically significant differences across groups are denoted by the “*p* < 0.05” notation. The letters on the plots for the flexural strength and flexural modulus indicate groups that show statistically significant differences in their measurements. Each group represents a specific type of CFRP with a different weight fraction of MWCNTs. The values of flexural strength and flexural modulus are also listed in Table 1.

The flexural strength results revealed that the reference sample (CFRP) without nanotubes had the lowest flexural strength compared with the samples containing different weight fractions of IL-treated P-MWCNTs and Ni-MWCNTs. According to the ANOVA of flexural strength, as shown in Figure 2a, the addition of IL-treated P-MWCNTs (CFRPIP) and Ni-MWCNTs (CFRPINi) improved flexural strength. Nevertheless, there was no significant difference among the samples containing MWCNTs, either P-MWCNTs or Ni-MWCNTs, in terms of the flexural strength properties, except for the CFRPIP-3.0 wt% sample, which showed the highest flexural strength represented by group “A”, which could be attributed to the enhanced sonication (45 min to 60 min) facilitating the dispersion of IL-treated P-MWCNTs (3.0 wt%) in the epoxy matrix, increasing the mechanical performance. Therefore, most of the MWCNTs-containing samples were statistically similar, as indicated by group “B”, while the CFRP sample showed a significant difference due to low flexural strength, as indicated by group “C”.

In terms of the flexural modulus (Figure 2b), CFRPs showed lower values compared with samples containing MWCNTs (CFRPIP and CFRPINi). Increasing the weight fractions of IL-treated P-MWCNTs in the CFRPIP series resulted in an increase in the flexural modulus, i.e., the stiffness of the hybrid CFRP laminates. A similar increase in flexural modulus was observed in the CFRPINi series containing various weight fractions of IL-treated Ni-MWCNTs. The CFRPIP-3.0 wt% sample exhibited the highest flexural modulus, while the CFRP sample showed a statistically significantly lower modulus than the others. Overall, the addition of nanotubes enhanced both the flexural strength and modulus, with the type and concentration affecting the degree of improvement.

**Figure 3:**
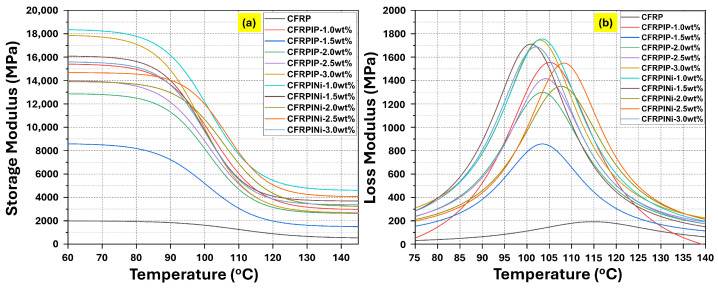
DMA graphs of hybrid nanocomposites (CFRPs): (**a**) storage modulus (MPa); (**b**) loss modulus (MPa).

**Figure 4:**
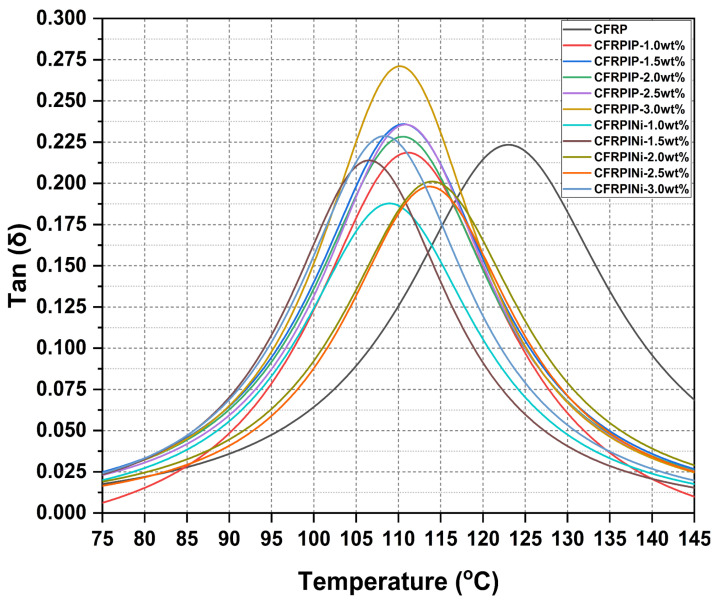
Tan δ graphs of hybrid nanocomposites (CFRPs).

Figure 3 and Figure 4 represent the dynamic mechanical analysis (DMA) test results for the hybrid CFRPs, indicating the effects of incorporating MWCNTs on the viscoelastic properties of these hybrid CFRPs. The reference CFRP sample demonstrated the lowest storage modulus (Figure 3a), while there was an increase in the modulus upon adding P-MWCNTs (CFRPIP samples), particularly at higher concentrations. Similarly, in the Ni-MWCNT (CFRPINi samples), the storage modulus increased but showed a decrease at higher concentrations, probably due to the enhanced interfacial bonding of the nickel coating with the epoxy matrix via the IL.

The loss modulus (Figure 3b) showed a significant increase with the addition of pristine carbon nanotubes, especially at higher concentrations, which could be attributed to the presence of more interfaces at the nano level. Ni-MWCNTs also enhanced the loss modulus, particularly at lower concentrations, which can be attributed to improved bonding and energy dissipation.

Tan δ (Figure 4), the ratio of the loss to storage modulus, represents the damping capability of the manufactured hybrid composites. Both P-MWCNTs and Ni-MWCNTs increased the tan δ peak, indicating improved vibration absorption, with this effect becoming more pronounced at higher MWCNT loading fractions. Therefore, incorporating both types of MWCNTs along with IL treatment improved the damping properties of CFRPs, with nickel-coated nanotubes potentially improving stiffness at higher concentrations. The variations in tan δ, which represent the damping capability of the CFRPs, are influenced by several mechanisms related to the incorporation of ionic liquid-treated MWCNTs. Firstly, the incorporation of IL-treated MWCNTs improves the interfacial bonding of MWCNTs with the epoxy matrix and carbon fibers. This enhanced bonding increases energy dissipation at the interfaces, leading to higher tan δ values, particularly at higher MWCNT concentrations. Secondly, the presence of MWCNTs introduces additional interfaces at the nanoscale within the hybrid CFRPs, which act as sites for energy absorption, further improving the damping capability of the composites. Thirdly, the type of MWCNT (pristine vs. nickel-coated) influences the molecular mobility within the epoxy matrix due to differences in aspect ratios and outer surfaces. Pristine MWCNTs, having cleaner surfaces and lower aspect ratios, place greater constraints on polymer chain mobility due to increased interfacial interactions, resulting in improved damping, whereas longer IL-treated nickel-coated MWCNTs may enhance stiffness but improve damping capacity to a lesser extent due to the nickel coating, high Ni content, longer lengths to form agglomerates, and reduced uniform dispersion, making them suitable for conductive structural applications due to their Ni content. These mechanisms collectively define the viscoelastic properties of the composites for specific applications.

In addition, the ionic liquid (IL) treatment of MWCNTs significantly affects the damping properties (tan δ), thermal stability, and glass transition temperature (Tg) of hybrid CFRPs. IL treatment enhances MWCNT dispersion in the epoxy matrix, improving interfacial interactions with carbon fibers and leading to increased energy dissipation, as shown by the higher tan δ peaks in composites with greater MWCNT concentrations. Cations and anions of IL promote non-covalent interactions with MWCNTs, which result in reduced agglomerates and enhance the number of energy-absorbing interfaces. In terms of thermal stability, IL treatment improves heat dissipation by preventing localized heat buildup due to MWCNTs that could degrade the composite. Moreover, IL treatment also affected the glass transition temperature (Tg) by affecting the molecular mobility within the epoxy matrix, inducing a plasticizing effect that lowered Tg (Table 2). Conversely, a reduced Tg enables the lower-temperature processing of hybrid nanocomposites. It could be concluded that by selecting appropriate nanomaterials and loading fractions, the viscoelastic properties of CFRPs could be tailored for specific applications.

**Figure 5:**
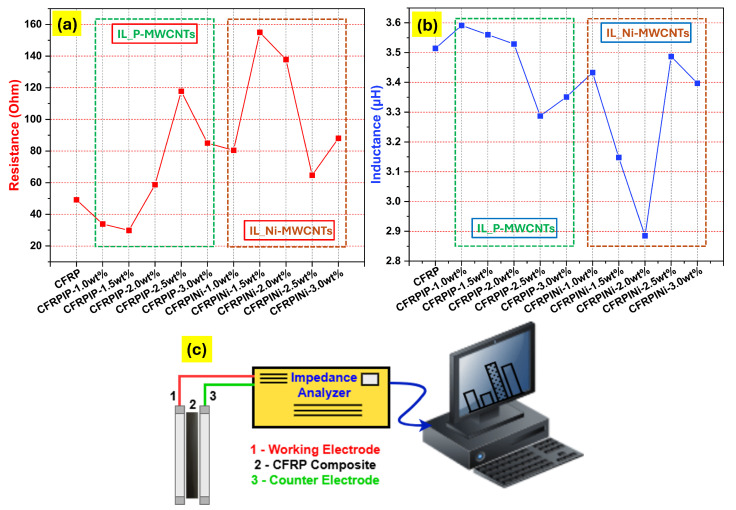
Impedance spectroscopy results for hybrid nanocomposites (CFRPs): (**a**) resistance; (**b**) inductance; (**c**) schematic diagram of impedance spectroscopy test.

Figure 5 illustrates the impedance spectroscopy results (i.e., resistance and inductance), as well as a schematic diagram of the impedance spectroscopy tests for the manufactured hybrid CFRPs, highlighting the effects of the type and concentration of MWCNTs on the electrical and magnetic properties of the hybrid CFRPs. As the weight percentage of MWCNTs increased, the electrical resistance (Figure 5a and Table 3) of the composite generally decreased, indicating improved conductivity. This is attributed to the formation of conductive pathways within the epoxy matrix, especially at 1.5 wt% of P-MWCNTs (CFRPIP-1.5 wt%). However, at higher concentrations, a reduction in conductivity was observed, which could be attributed to the formation of agglomerates of P-MWCNTs. Ni-MWCNTs tend to exhibit higher resistance than P-MWCNTs at the same weight fractions. This phenomenon could be attributed to the nickel coating on the surface of MWCNTs, which initially impedes electron flow, or to the reduced length of Ni-MWCNTs compared with P-MWCNTs, leading to lower availability of conducting pathways. Therefore, the percolation threshold value was observed at higher loading fractions of Ni-MWCNTs (CFRPINi-2.5 wt%).

The inductance value (Figure 5b) showed pronounced changes with the addition of both types of IL-treated MWCNTs. However, IL-treated Ni-MWCNTs showed more evident changes in inductance due to the ferromagnetic nature of nickel, which enhanced the magnetic properties of the composite. Moreover, the use of IL treatment, particularly $NTf_{2}$, improved the dispersion of MWCNTs within the epoxy matrix, resulting in more effective conductive pathways, and enhanced interfacial bonding between the MWCNTs and the epoxy matrix, leading to further improvements in the electrical performance of composites. Therefore, the choice between P-MWCNTs and Ni-MWCNTs depends on the requirements of the desired application. P-MWCNTs offer higher electrical conductivity, which makes them suitable for low-resistance applications. Conversely, Ni-MWCNTs are preferred where enhanced magnetic properties are of major concern, like in sensors or EMI-shielding applications.

**Figure 6:**
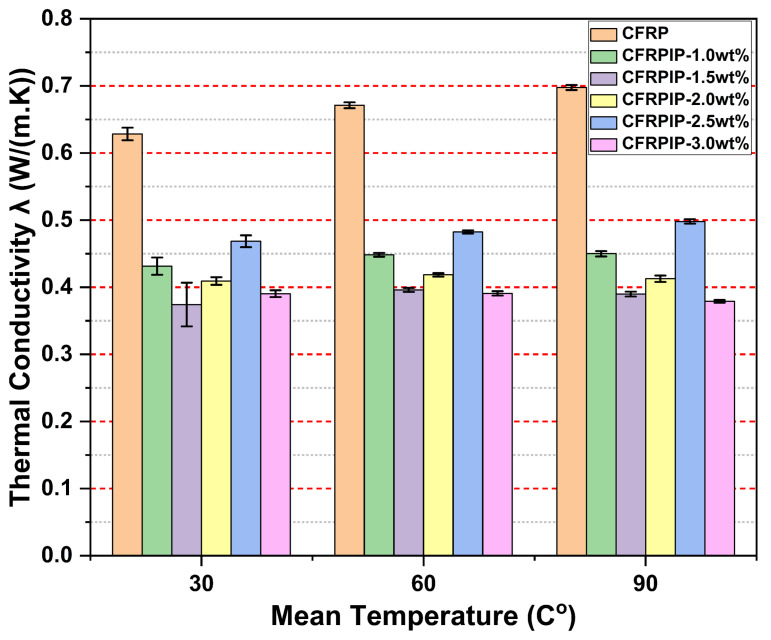
Thermal conductivity test results for CFRPs containing IL-treated pristine MWCNTs.

**Figure 7:**
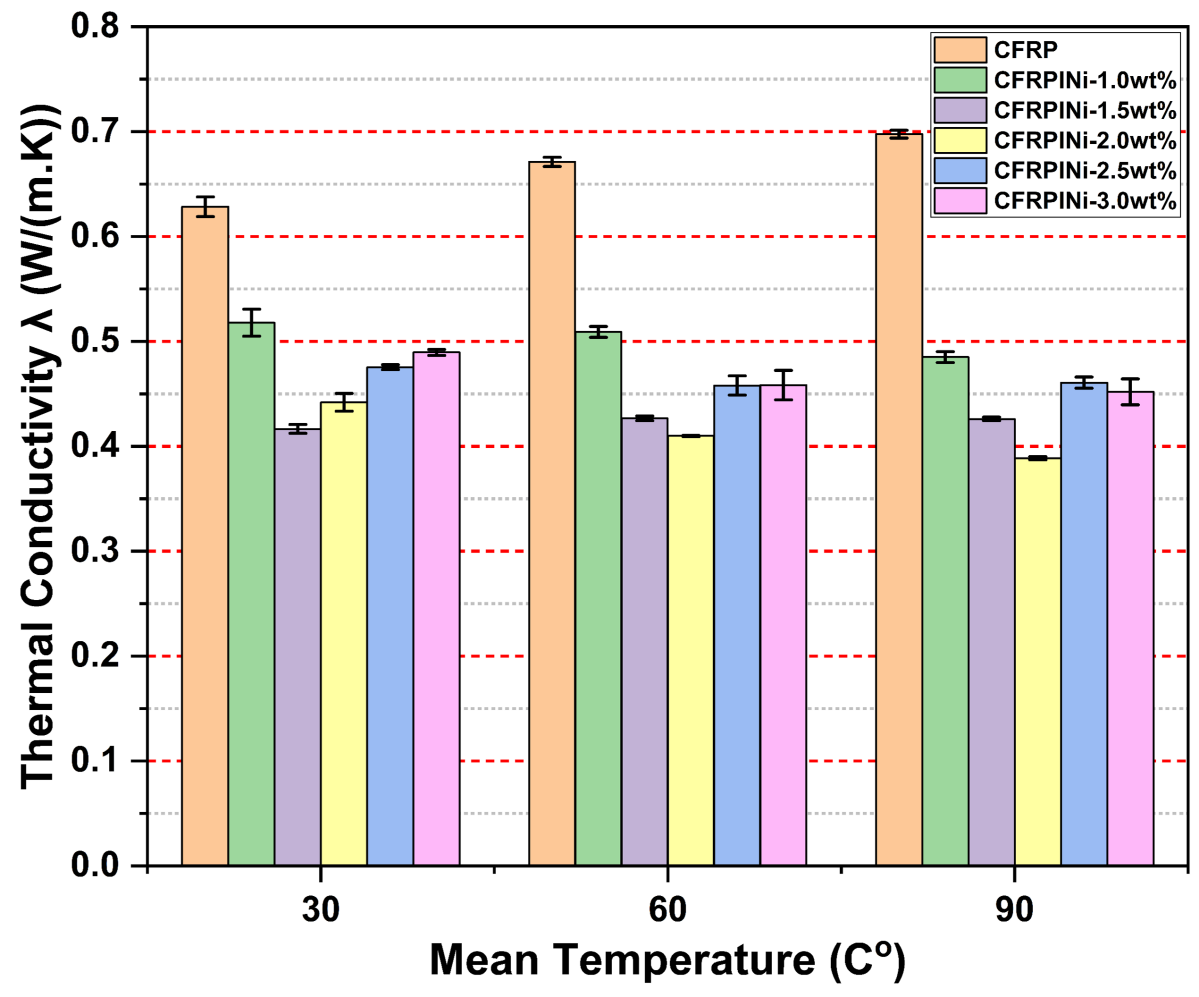
Thermal conductivity test results for CFRPs containing IL-treated Ni-coated MWCNTs.

Figure 6 and Figure 7 represent the thermal conductivity (TC) test results for the manufactured composites. CFRP, the reference sample, comprising carbon fibers and the epoxy matrix without MWCNTs, showed an increase in thermal conductivity as the temperature was raised, from 0.6284 W/(m · K) at 30 °C to 0.69772 W/(m · K) at 90 °C. This behavior is consistent with the fact that materials conduct heat better at higher temperatures. Further, the presence of carbon fibers in the epoxy matrix assists in conducting heat, while the epoxy helps to retain the structure.

In contrast, the CFRPIP samples (Figure 6), containing various weight fractions of P-MWCNTs treated with ionic liquid, showed lower thermal conductivity than CFRP. For example, CFRPIP-1.0wt% had a thermal conductivity of 0.43139 W/(m · K) at 30 °C, significantly lower than that of CFRP. Even as the temperature increased, the thermal conductivity of the CFRPIP samples remained low compared with that of CFRP. The lower thermal conductivity performance might be due to the absorption of heat by MWCNTs, which makes carbon fibers able to dissipate the heat from the bulk of the composite. Adding more MWCNTs (e.g., 1.5 wt% and 2.0 wt%) could not improve the TC, suggesting that the MWCNTs serve as heat sinks within the epoxy matrix, thereby absorbing the incoming heat flux and slowing down the dissipation of heat.

The CFRPINi samples (Figure 7), which contained Ni-MWCNTs treated with ionic liquid, performed slightly better than CFRPIP but still worse than CFRP. For instance, CFRPINi-1.0wt% had a thermal conductivity of 0.5179 W/(m · K) at 30 °C, higher than that of CFRPIP-1.0wt% but lower than that of CFRP. As the temperature increased, the thermal conductivity of the CFRPINi samples either remained the same or decreased slightly. The nickel coating might have been the reason for the less effective thermal response compared with that of P-MWCNTs because, while theoretically improving heat conduction, it has a shorter length than the P-MWCNTs. Therefore, even higher nanotube concentrations (e.g., 2.0 wt%) did not significantly improve performance, with CFRPINi-2.0wt% reaching only 0.44196 W/(m · K) at 30 °C, still much lower than that of CFRP.

The addition of P-MWCNTs and Ni-MWCNTs to the epoxy reduced its thermal conductivity compared with CFRP. This unexpected behavior suggests that the interaction between the nanotubes and the epoxy matrix plays a crucial role in determining the overall thermal properties of the composites. However, the use of these properties is important in sectors such as battery packaging and electrical insulation. In such applications, effective thermal management is needed to maintain optimal performance as well as to ensure the safety of the devices under extreme conditions. The use of these hybrid CFRPs in these applications could serve to significantly enhance safety measures.

**Figure 8:**
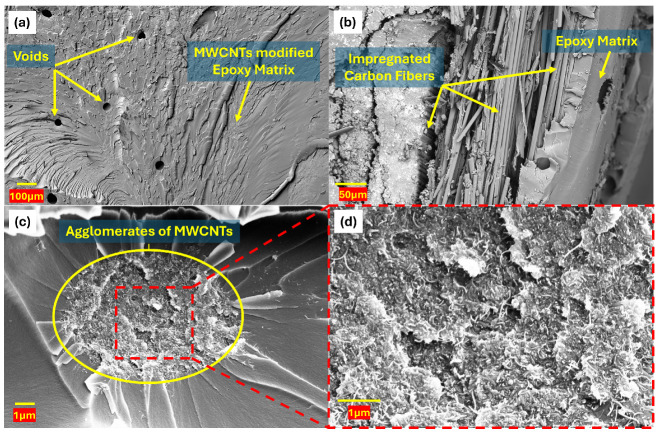
SEM images of hybrid nanocomposites (CFRPs): (**a**) microvoids in CFRPINi-3.0wt% (**b**) epoxy-impregnated carbon fibers in CFRPINi-1.5wt%; (**c**) agglomerates of MWCNTs in CFRPIP-2.5wt%; (**d**) magnified view of agglomerates of MWCNTs.

Figure 8 shows four SEM images highlighting the microstructure of the hybrid CFRPs. Figure 8a shows a hybrid CFRP with 3 wt% ionic liquid-treated nickel-coated MWCNTs (CFRPINi-3.0wt%), revealing voids in the epoxy matrix, likely due to the enhanced viscosity of the modified resin leading to the incomplete wetting of carbon fibers and/or entrapped air bubbles during fabrication through the vacuum bagging technique. These voids may reduce mechanical strength and electrical conductivity by creating stress concentration sites. Figure 8b displays a CFRP with carbon fibers well impregnated by modified epoxy resin, indicating strong interfacial bonding, which is essential for improving the load-bearing capabilities of manufactured composites through efficient stress transfer. Figure 8c depicts a CFRP with 2.5 wt% ionic liquid-treated pristine MWCNTs (CFRPIP-2.5wt%), showing MWCNT agglomerates due to the non-uniform dispersion of MWCNTs, ultimately leading to decreases in the mechanical and electrical performance of the manufactured hybrid CFRPs. Figure 8d, a magnified view of Figure 8c, reveals an entangled MWCNT network within agglomerates, which could probably act as voids and/or stress concentration sites instead of offering localized reinforcement at the nanoscale. These SEM images explain the microstructural variations due to MWCNTs’ type and their weight fractions, explaining differences in the mechanical, electrical, and thermal properties of manufactured hybrid CFRPs by suggesting an improved dispersion of MWCNTs in the matrix, which could optimize battery pack applications.

**Figure 9:**
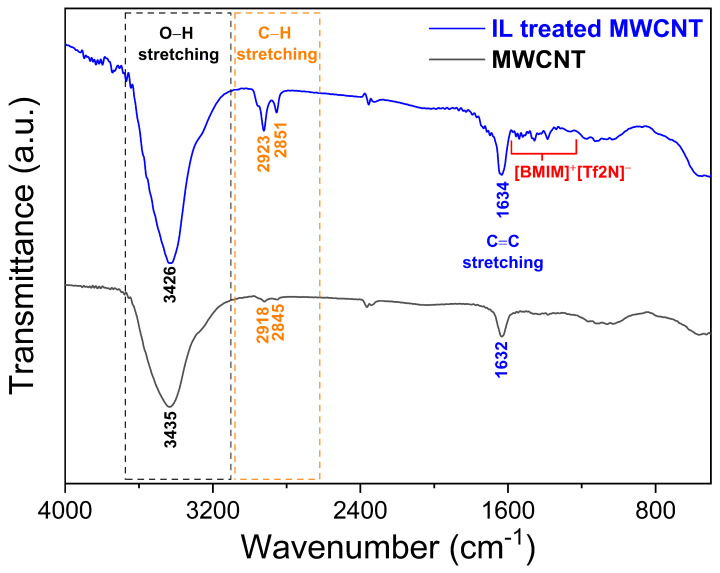
FTIR results verifying the presence of ionic liquid on the surface of MWCNTs.

The FTIR analysis (Figure 9) of functionalized multiwalled carbon nanotubes (MWCNTs) revealed key functional groups and bonds introduced during modification. A strong, broad peak at 3426 cm^−1^ confirmed the presence of hydroxyl (-OH) groups, likely from residual water or hydroxylation during functionalization. Weak C–H stretching vibrations at $2923\;{\mathrm{cm}}^{-1}$ and $2851\;{\mathrm{cm}}^{-1}$ suggested alkyl chain incorporation, potentially from the butyl group of the [BMIM][Tf_2_*N*] ionic liquid. A very weak peak at $1733\;{\mathrm{cm}}^{-1}$ hinted at minor oxidation (C=O), though its low intensity implies limited carbonyl formation. The $1634\;{\mathrm{cm}}^{-1}$ peak corresponded to C=C aromatic stretching in the MWCNT framework, consistent with the graphitic structure.

Peaks at $1390\;{\mathrm{cm}}^{-1}$, at $1457\;{\mathrm{cm}}^{-1}$, and in the 1500–1600 $\;{\mathrm{cm}}^{-1}$ range were attributed to imidazolium ring vibrations (from ${\left[\mathrm{BMIM}\right]}^{+}$) and C–H bending in alkyl chains, confirming ionic liquid interaction. Notably, the expected S–N–S stretching modes of the ${\left[{\mathrm{Tf}}_{2}N\right]}^{-}$ anion (1350–1200 ${\mathrm{cm}}^{-1}$) were absent, implying weak or non-covalent interactions with MWCNTs. The anomalous doublet at $2368/2334\;{\mathrm{cm}}^{-1}$ was likely overtones or artifacts rather than fundamental bonds. Overall, the spectrum validated successful hydroxylation and alkyl chain functionalization, with evidence of ${\left[\mathrm{BMIM}\right]}^{+}$ cation integration but minimal covalent bonding with the ${\left[{\mathrm{Tf}}_{2}N\right]}^{-}$ anion. These findings align with surface modifications typical of ionic liquid-treated MWCNTs, where physical adsorption or electrostatic interactions dominate over covalent bonding for the anion. The absence of strong oxidation signals (e.g., pronounced C=O) suggests controlled functionalization, preserving the structural integrity of the nanotubes while introducing desired surface groups for enhanced compatibility or reactivity.

## 4. Conclusions

Treating P-MWCNTs and Ni-MWCNTs with BMI-TFSI significantly improves the properties of these nanomaterials, which ultimately enhances the electrical, mechanical, and thermal properties of the bulk composites. Ionic liquid facilitates proper dispersion by increasing the interaction of MWCNTs with the epoxy matrix due to cations and anions. For Ni-MWCNTs (with >60 wt% nickel), the higher nickel content in nickel-coated MWCNTs further improves the electrical and thermal conductivity of MWCNTs, while BMI-TFSI ionic liquid enhances the toughness and impact resistance by improving dispersion and interfacial bonding. As a result, these modifications lead to enhanced mechanical properties, including increased strength and durability, while also optimizing the composite’s thermal stability and electrical performance. This combination of improved properties makes these hybrid nanocomposites highly suitable for advanced applications in electronics, aerospace, and automotive industries. Moreover, their low thermal conductivity and high thermal resistance could be beneficial from a fire safety perspective. This characteristic may help to prevent the rapid spread of flames and heat, offering valuable time for evacuation and minimizing damage in the case of fire hazards. This makes the material particularly suitable for applications where thermal management is critical to ensure safety and performance under extreme conditions, as in battery packaging and electrical insulation. The integration of hybrid CFRPs in these applications could not only enhance safety aspects but may also contribute to increasing the lifespan and efficiency of electronic devices.

## Figures and Tables

**Table 1:** Flexural test results for hybrid nanocomposites (CFRPs).

Sample ID	Flexural Strength (MPa)	S.D.	Flexural Modulus (GPa)	S.D.
CFRP	319	35	36.6	1.5
CFRPIP-1.0wt%	668.7	49.3	56.89	1.2
CFRPIP-1.5wt%	637.1	67.2	67.71	1.7
CFRPIP-2.0wt%	667.8	63.7	61.78	7.6
CFRPIP-2.5wt%	648.5	40.2	73.67	3.7
CFRPIP-3.0wt%	802.8	67.7	88.21	1
CFRPINi-1.0wt%	655	44.6	52.41	6.8
CFRPINi-1.5wt%	588.5	28.4	55.67	1.3
CFRPINi-2.0wt%	597.5	41.8	61.05	1.9
CFRPINi-2.5wt%	610.3	44.2	58.29	1.7
CFRPINi-3.0wt%	593.2	39.5	68.13	3.7

**Table 2:** Glass transition (Tg) temperature values of hybrid nanocomposite (CFRPs).

Sample ID	Tg (°C)
CFRP	123
CFRPIP-1.0wt%	111
CFRPIP-1.5wt%	110
CFRPIP-2.0wt%	110
CFRPIP-2.5wt%	110
CFRPIP-3.0wt%	110
CFRPINi-1.0wt%	108
CFRPINi-1.5wt%	106
CFRPINi-2.0wt%	113
CFRPINi-2.5wt%	113
CFRPINi-3.0wt%	108

**Table 3:** Impedance spectroscopy results for hybrid nanocomposites (CFRPs).

Sample ID	$R_{1}$	%Error	$L_{1}$	%Error	Equivalent Circuit
CFRP	49.2	0.069	0.3514 $\times \;10^{-5}$	0.764	$R_{1}$ $L_{1}$
CFRPIP-1.0wt%	33.9	0.055	0.3591 $\times \;10^{-5}$	0.426	$R_{1}$ $L_{1}$
CFRPIP-1.5wt%	29.89	0.058	0.3560 $\times \;10^{-5}$	0.410	$R_{1}$ $L_{1}$
CFRPIP-2.0wt%	58.7	0.067	0.3529 $\times \;10^{-5}$	0.861	$R_{1}$ $L_{1}$
CFRPIP-2.5wt%	117.8	0.084	0.3287 $\times \;10^{-5}$	2.237	$R_{1}$ $L_{1}$
CFRPIP-3.0wt%	85.0	0.078	0.3351 $\times \;10^{-5}$	1.482	$R_{1}$ $L_{1}$
CFRPINi-1.0wt%	80.5	0.075	0.3433 $\times \;10^{-5}$	1.318	$R_{1}$ $L_{1}$
CFRPINi-1.5wt%	155.0	0.087	0.3148 $\times \;10^{-5}$	3.149	$R_{1}$ $L_{1}$
CFRPINi-2.0wt%	137.8	0.089	0.2885 $\times \;10^{-5}$	3.120	$R_{1}$ $L_{1}$
CFRPINi-2.5wt%	64.7	0.071	0.3487 $\times \;10^{-5}$	1.010	$R_{1}$ $L_{1}$
CFRPINi-3.0wt%	88.1	0.079	0.3397 $\times \;10^{-5}$	1.543	$R_{1}$ $L_{1}$

## Data Availability

Data are contained within the article.
